# Presence of immunogenic alternatively spliced insulin gene product in human pancreatic delta cells

**DOI:** 10.1007/s00125-023-05882-y

**Published:** 2023-03-08

**Authors:** René van Tienhoven, Maria J. L. Kracht, Arno R. van der Slik, Sofia Thomaidou, Anouk H. G. Wolters, Ben N. G. Giepmans, Juan Pablo Romero Riojas, Michael S. Nelson, Françoise Carlotti, Eelco J. P. de Koning, Rob C. Hoeben, Arnaud Zaldumbide, Bart O. Roep

**Affiliations:** 1 Department of Diabetes and Cancer Discovery Science, Arthur Riggs Diabetes & Metabolism Research Institute, Beckman Research Institute, City of Hope National Medical Center, Duarte, CA USA; 2 Department of Cell and Chemical Biology, Leiden University Medical Center, Leiden, the Netherlands; 3 Department of Immunology, Leiden University Medical Center, Leiden, the Netherlands; 4 Department of Biomedical Sciences of Cells and Systems, University Medical Center Groningen, University of Groningen, Groningen, the Netherlands; 5 Department of Internal Medicine, Leiden University Medical Center, Leiden, the Netherlands; 6 Light Microscopy Core, City of Hope National Medical Center, Duarte, CA USA

**Keywords:** Alternative splicing, Alternative translation, Beta cell, Defective ribosomal product, Delta cell, Human islets of Langerhans, Insulin gene, Insulin-degrading enzyme, Type 1 diabetes

## Abstract

**Aims/hypothesis:**

Transcriptome analyses revealed insulin-gene-derived transcripts in non-beta endocrine islet cells. We studied alternative splicing of human *INS* mRNA in pancreatic islets.

**Methods:**

Alternative splicing of insulin pre-mRNA was determined by PCR analysis performed on human islet RNA and single-cell RNA-seq analysis. Antisera were generated to detect insulin variants in human pancreatic tissue using immunohistochemistry, electron microscopy and single-cell western blot to confirm the expression of insulin variants. Cytotoxic T lymphocyte (CTL) activation was determined by MIP-1β release.

**Results:**

We identified an alternatively spliced *INS* product. This variant encodes the complete insulin signal peptide and B chain and an alternative C-terminus that largely overlaps with a previously identified defective ribosomal product of *INS*. Immunohistochemical analysis revealed that the translation product of this *INS*-derived splice transcript was detectable in somatostatin-producing delta cells but not in beta cells; this was confirmed by light and electron microscopy. Expression of this alternatively spliced *INS* product activated preproinsulin-specific CTLs in vitro. The exclusive presence of this alternatively spliced *INS* product in delta cells may be explained by its clearance from beta cells by insulin-degrading enzyme capturing its insulin B chain fragment and a lack of insulin-degrading enzyme expression in delta cells.

**Conclusions/interpretation:**

Our data demonstrate that delta cells can express an *INS* product derived from alternative splicing, containing both the diabetogenic insulin signal peptide and B chain, in their secretory granules. We propose that this alternative *INS* product may play a role in islet autoimmunity and pathology, as well as endocrine or paracrine function or islet development and endocrine destiny, and transdifferentiation between endocrine cells. *INS* promoter activity is not confined to beta cells and should be used with care when assigning beta cell identity and selectivity.

**Data availability:**

The full EM dataset is available via www.nanotomy.org (for review: http://www.nanotomy.org/OA/Tienhoven2021SUB/6126-368/). Single-cell RNA-seq data was made available by Segerstolpe et al [13] and can be found at https://sandberglab.se/pancreas. The RNA and protein sequence of INS-splice was uploaded to GenBank (BankIt2546444 INS-splice OM489474).

**Graphical abstract:**

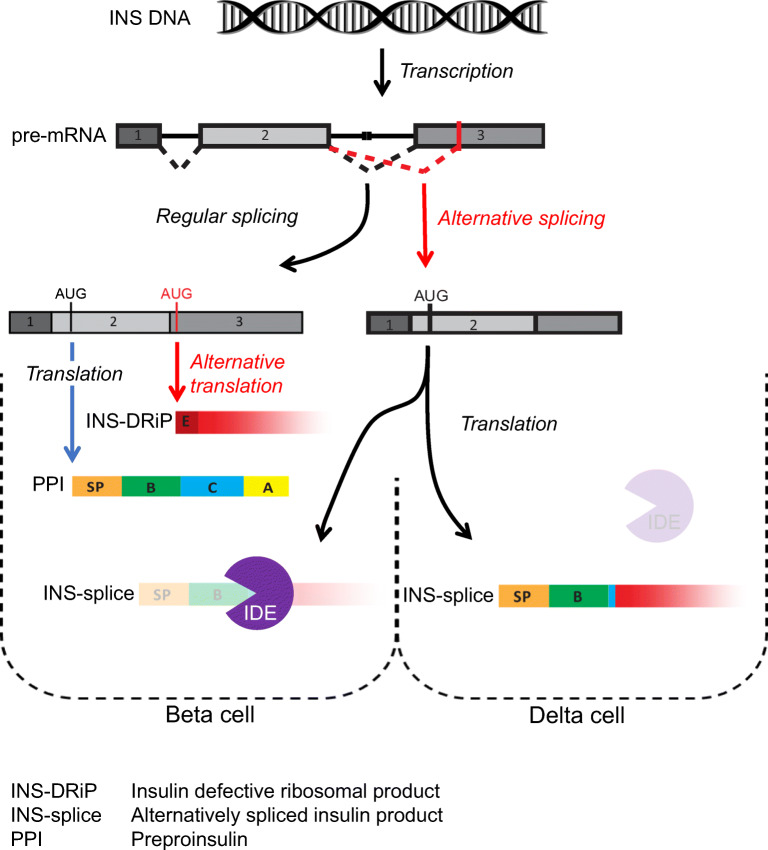

**Supplementary Information:**

The online version of this article (10.1007/s00125-023-05882-y) contains peer-reviewed but unedited supplementary material..

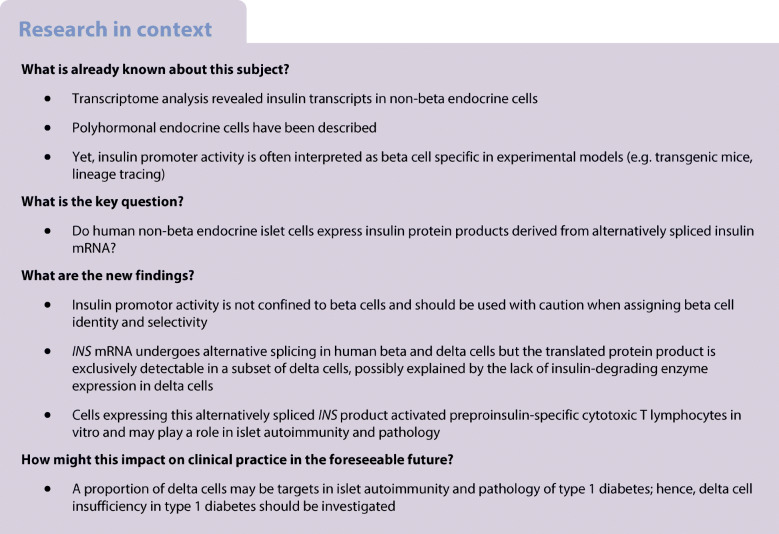



## Introduction

Polyhormonal endocrine cells have been shown to reside in human fetal pancreatic islets and in individuals with type 2 diabetes and chronic pancreatitis but fully differentiated endocrine cells are classically dedicated to produce a single hormone (i.e. glucagon by alpha cells, insulin by beta cells and somatostatin by delta cells) [2–5]. Under this definition, insulin gene (*INS*) expression is restricted to pancreatic beta cells. Yet, accumulating data indicate that even mature human beta cells are more plastic than previously assumed [6]. While the differentiated state of beta cells is maintained by reinforcement of specific gene regulatory networks and repression of other transcriptional programmes [7–10], specific circumstances such as metabolic and mechanical stress have been shown to cause spontaneous dedifferentiation and transdifferentiation of human beta cells [11, 12]. Conversion of beta cells into alpha and delta cell-like states observed in individuals with type 2 diabetes has been proposed to contribute to reduced functional beta cell mass and beta cell failure [3]. Furthermore, in vitro disruption of human islet integrity has been reported to cause the spontaneous conversion of some beta cells into glucagon-producing cells. This endocrine plasticity has been proposed to allow dysfunctional beta cells to escape apoptosis due to environmental stress as well as replenish beta cell mass [13]. In situ hybridisation and single-cell transcriptome analysis of human islet cells have confirmed the presence of *INS* mRNA in alpha and delta cells [1]. Approximately 46% of islet cells were found to express more than one additional hormonal transcript per cell, with a considerable portion containing both insulin and somatostatin transcripts [14].

Alternative splicing increases proteome diversity by generating multiple mRNA transcripts from a single gene that differ in their assembly of exons and introns. Approximately 95% of the human transcriptome is estimated to derive from alternatively spliced transcripts [15]. Tissue-specific splicing patterns allow expression of genes in different cell types to produce protein isoforms that differ in biological composition and activity [16]. Alternative splicing networks are implicated in a broad variety of biological processes, including; maintenance of pluripotency, directing cell differentiation, cell lineage commitment and tissue identity [17]. Splicing patterns are highly dynamic and therefore provide a mechanism to allow swift adaptation to changes in the local microenvironment [18]. Many tumours undergo alternative splicing, potentially generating neoantigens that are prominent targets in cancer immunotherapy [19]. Likewise, splice variants generated in beta cells may contribute to autoimmunity and type 1 diabetes [20, 21]. Type 1 diabetes autoimmunity is hallmarked by insulitis in which beta cells are specifically destroyed by autoreactive cytotoxic T lymphocytes (CTLs) that are highly reactive to preproinsulin (PPI) epitopes [22–24]. The beta cell transcriptome was shown to be highly impacted by inflammatory and metabolic insults [25]. Long RNA sequencing and ribosomal profiling revealed the extreme diversity of the beta cell transcriptome and proteome [26]. Experiments conducted in HEK293T cells overexpressing *INS* demonstrated the presence of cryptic splice sites in *INS* mRNA, as multiple PPI-coding insulin transcript variants were detected [27, 28].

We investigated alternative splicing of *INS* mRNA in human islets and determined the expression and immunogenicity of alternative insulin protein products in endocrine cells.

## Methods

### Human islets

Pancreatic islets were obtained from human cadaveric donor pancreases with consent. The reported investigations were carried out in accordance with the declaration of Helsinki (2008). Islets were isolated as previously described [29]. See electronic supplementary material (ESM) Methods for details. The checklist for reporting human islet preparations is presented in ESM Table 1.

### Cell culture

HEK293T cells (ATCC CRL-3216) were maintained in high-glucose DMEM (Gibco-BRL, Breda, the Netherlands) supplemented with 8% FBS (Gibco-BRL), 100 units/ml penicillin and 100 μg/ml streptomycin (Gibco-BRL). Cells were mycoplasma negative.

### DNA constructs and transfection

Insulin-expressing vectors were cloned using human genomic DNA and verified by Sanger sequencing. HEK293T cells were transfected using polyethylenimine and harvested 48 h post transfection. See ESM Methods for details.

### Western blotting

Standard western blotting protocols were followed using HEK293T cell lysate and antibodies against insulin, C-peptide, actin, GFP, SPLICE_81-95_ and somatostatin. See ESM Methods for details.

### Flow cytometry

Human islets were dispersed into single cells, fixed, permeabilised, stained with SPLICE_81-95_ antiserum and analysed using FACS Aria II (BD Biosciences, USA). See ESM Methods for details.

### ELISA

Plates were coated with recombinant polypeptide and blocked with 2% BSA, followed by incubation with antibodies against DRiP_1-13_, SPLICE_81-95_ or C-peptide. Antibodies were visualised with horseradish peroxidase (HRP)-conjugated secondary antibodies and HRP substrate. Absorbance was measured at 450 nm. See ESM Methods for details.

### Electron microscopy

Electron microscopy (EM) islet datasets were created from nPOD donors. Sections (80 nm) were placed on formvar-coated copper grids and contrasted with uranyl acetate. Sections were immunolabelled with gold or quantum dots, using SPLICE_81-95_ antiserum. Data were acquired by Supra 55 scanning EM (SEM; Zeiss, Oberkochen, Germany) using a scanning transmission EM detector at 28 kV with 2.5 nm pixel size and an external scan generator ATLAS 5 (Fibics, Ottawa, ON, Canada). See ESM Methods for details.

### Single-cell transcriptome analysis

Single-cell RNA-seq data from non-diabetic donors were acquired online [1]. Data from all delta and beta cells were merged into single BAM files per donor. Reads in the region of interest (chr11:2157102-2163862) were extracted and Sashimi plots were generated using the Integrative Genomics Browser.

### Generation of custom polyclonal antisera

Custom polyclonal antisera were generated by immunising rabbits with the synthetic peptides MLYQHLLPLPAGEC (DRiP_1-13_; cysteine served as anchor residue for the carrier) and LLHRERWNKALEPAK (SPLICE_81-95_) (Eurogentec, Belgium). The rabbits were repeatedly boosted for 28 days with synthetic peptide and bled before and after immunisation. Immune reactivity to the specific peptides was tested by ELISA performed by the manufacturer.

### Immunohistochemistry and microscopy

Paraffin-embedded human tissues were cut into 4 μm sections, deparaffinised in xylene and rehydrated. Antigen retrieval was performed prior to staining with antibodies against insulin, C-peptide, glucagon, somatostatin, DRiP_1-13_, SPLICE_81-95_, insulin B chain, insulin-degrading enzyme (IDE) and proinsulin. Immunofluorescence was detected with a Leica SP8 (Leica, Germany) or Zeiss LSM880 confocal microscope. Manders co-localisation coefficients (MCCs) were determined using QuPath 0.2.3 [30]; the analysis script is available in the ESM (ESM QuPath Colocalisation Script). See ESM Methods for details.

### Single-cell western blot

Human islets were dispersed into single cells using trypsin and filtration. Single islet cells were loaded onto scWest chips (Protein Simple, San Jose, CA, USA), then lysed, electrophoresed and UV-crosslinked according to the manufacturer’s protocol. Chips were probed with primary antibodies against somatostatin (1:30, A0566; Dako, Denmark), insulin B chain (1:10, M093-3; MBL, USA) and insulin C-peptide (1:15, ab14181; Abcam, UK). The appropriate Alexa-conjugated secondary antibodies were used. DNA was stained with Yoyo1 (Invitrogen, USA). Immunofluorescence was detected with the GenePix 4400A microarray scanner (Molecular Devices, USA) at 2.5 μm resolution.

### Recombinant polypeptides

Human recombinant polypeptides were synthesised as previously described [31]. Protein encoding cDNA was obtained from human pancreatic islets by PCR and cloned in pDest17 for protein production in *Escherichia coli* using gateway cloning technology (Invitrogen, Carlsbad, CA, USA). Recombinant proteins were purified by His6 affinity purification tag and freeze-dried. Purified polypeptides were dissolved in 0.05% acetic acid in MQ/PBS to a stock concentration of 1 mg/ml.

### CTL activation assay

HEK293T cells expressing the alternatively spiced *INS* mRNA were cocultured with CTLs directed against the PPI signal peptide PPI_15-24_. The supernatant fraction was used for detection of MIP-1β production by the CTLs. See ESM methods for details.

### IDE cleavage assay

Recombinant alternatively spliced insulin product (INS-splice; 0.25 μg/μl) was incubated with recombinant human IDE (0.05 μg/μl, 2496-ZN; R&D Systems, USA) in cleavage buffer (50 mmol/l Tris, 1 mol/l NaCl, pH 7.5) at 37°C for 48 h, following the manufacturer’s recommendations. Samples were heat-inactivated at 70°C for 5 min, diluted in LDS sample buffer (Thermo Fisher Scientific, USA) and heated at 70°C for 10 min before loading onto a 12% Bis-Tris gel (Thermo Fisher Scientific). Gel was stained with Coomassie blue (Bio-Rad, USA) according to the manufacturer’s protocol.

### Statistical analysis

All data points are presented as mean values (±SD). Statistical calculations were carried out using Graphpad Prism 9 (Graphpad software, San Diego, CA, USA). Statistical tests are indicated in the figure legends. A *p* value of <0.05 was considered significant.

## Results

### Evidence of alternative *INS* RNA splicing in human islets

**Fig. 1:**
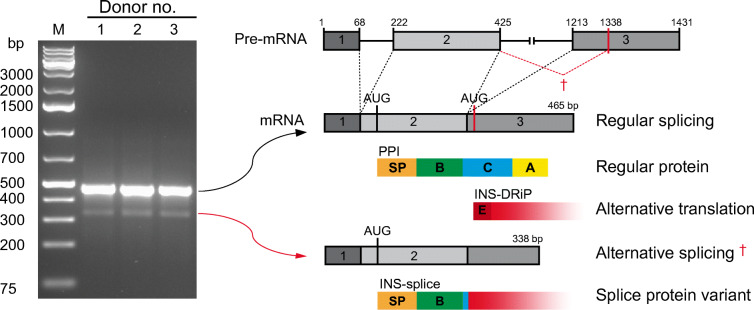
Alternative *INS* RNA splicing in human islets. Analysis of *INS* splicing by PCR on RNA derived from human pancreatic islets of three different donors, visualised on DNA gel. A schematic overview of the human insulin pre-mRNA is shown with the exons annotated by numbers (1–3) and the intronic regions represented by a black solid line. Normal *INS* mRNA splicing and alternative *INS* mRNA splicing are indicated by black and red dashed lines, respectively (showing the start codon, AUG). The resulting mRNA products with translation initiation sites are depicted underneath. For each mRNA molecule the potential protein products are displayed. Regular protein translation of the regular spliced transcript produces preproinsulin (PPI) with the signal peptide (orange), B chain (green), C-peptide (blue) and insulin A chain (yellow). Alternative translation of this transcript produces INS-DRiP, with the previously identified CTL epitope (dark red). Translation of the alternatively spliced transcript produces a splice protein variant, referred to as INS-splice. Amino acid sequences are indicated with corresponding colours and letters indicate the presence of the complete chain. The non-stop characteristic of INS-DRiP and INS-splice proteins is visualised by decreasing gradient. A, insulin A chain; B, insulin B chain; C, C-peptide; E, CTL epitope; M, DNA marker; INS, insulin; SP, signal peptide

Analyses performed on RNA isolated from human islets of three different donors identified two major *INS* RNA variants (Fig. 1). Nucleotide sequencing of these *INS* cDNA variants indicated that the larger, more-abundant *INS* RNA variant represents full-length PPI in which intron 1 and 2 have been fully spliced out (ESM Fig. 1). This full-length *INS* mRNA has been shown to generate an insulin defective ribosomal product (INS-DRiP), in particular under endoplasmic reticulum (ER) stress, which is a target of islet autoimmunity and associated with type 1 diabetes pathology [32]. The shorter, less-abundant cDNA variant resulted from a cryptic splicing site within exon 3 at position 1338 (ESM Fig. 1), predicted by in silico analysis (not shown). The open reading frame that is formed by this alternative splicing may lead to the translation of a polypeptide in which the signal peptide and B chain of the canonical PPI are intact but the C-terminal end of the molecule differs because of RNA translation into the +2 reading frame (referred to as INS-splice). Coincidently, this C-terminal region is identical to the C-terminus of INS-DRiP except for the first ten-amino-acid immunodominant N-terminus that is unique to INS-DRiP [32] (Fig. 1).

### Alternatively spliced *INS* mRNA is a template for translation in delta cells

To investigate these alternative *INS*-derived proteins, rabbits were immunised with a short polypeptide unique to INS-DRIP (DRiP_1-13_) and a short polypeptide of the C-terminus shared between INS-DRiP and the predicted polypeptide INS-splice (‘SPLICE_81-95_’). The peptides were selected from analysis of the UniProt human protein Knowledgebase using the basic local alignment search tool (BLAST) to avoid cross-reactivity to other known proteins (data not shown). Antiserum specificity was confirmed by ELISA using recombinant PPI, INS-DRiP and INS-splice (ESM Fig. 2). As expected, neither antisera cross-reacted with PPI. While the anti-DRiP_1-13_ antiserum specifically detected the INS-DRiP polypeptide recognised by cytolytic T cells in individuals with type 1 diabetes, the anti-SPLICE_81-95_ antiserum reacted with both recombinant INS-DRiP and INS-splice proteins (ESM Fig. 2).

**Fig. 2:**
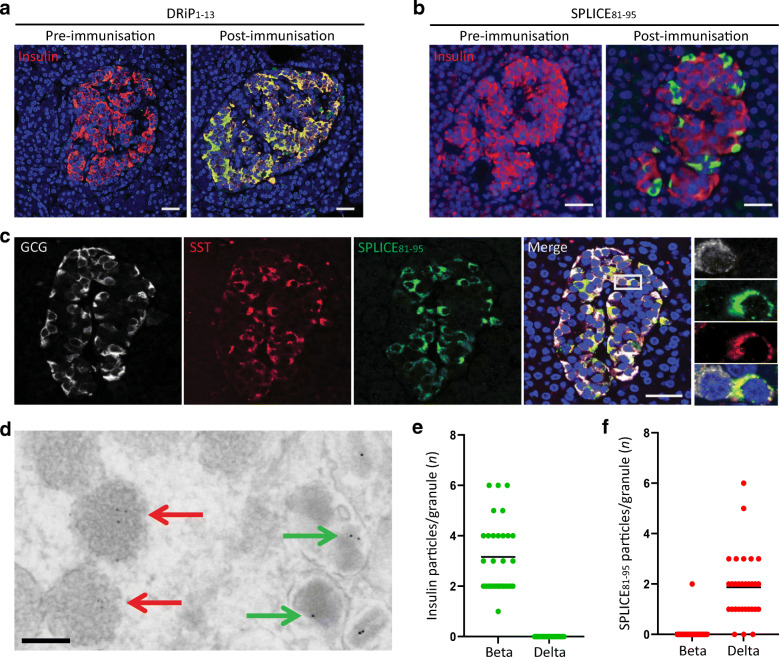
SPLICE_81-95_ antiserum stained delta cells and INS-splice protein is localised to somatostatin granules. (**a**, **b**) Immunohistochemistry of human pancreas sections with pre-immunisation serum and post-immunisation serum (green) in combination with insulin (red). Serum derived from DRiP_1-13_ immunised rabbits (**a**) and SPLICE_81-95_ immunised rabbits (**b**) was used. Scale bar, 30 μm. (**c**) Human pancreas sections stained for glucagon (white) and somatostatin (red), and SPLICE_81-95_ antiserum (green). Enlarged images of the grey enclosure are shown. Nuclei were visualised by DAPI staining (blue). Scale bar, 30 μm. (**d**) EM images of human pancreas sections labelled for INS-splice (quantum dots, red arrows) and insulin (immunogold, green arrows) visible as black dots. Scale bar, 200 nm. The granules were identified by their morphology. The full dataset is available via www.nanotomy.org (for review, see http://www.nanotomy.org/OA/Tienhoven2021SUB/6126-368/). (**e**, **f**) Quantification of the insulin-immunogold^+^ (**e**) and INS-splice-quantum dot^+^ (**f**) granules in beta and delta cells. Each granule is represented as a point. The graphs represent the means of 30 beta and 30 delta cell granules

To investigate whether the *INS*-derived polypeptides are generated in islets, human pancreatic sections were stained with the pre-immunisation or post-immunisation antisera. The localisation of the N-terminal INS-DRiP polypeptide within beta cells is consistent with our previous findings and supports beta cell destruction by CTLs directed against INS-DRiP [32] (Fig. 2a). Yet, the SPLICE_81-95_ antiserum raised to the C-terminus shared between INS-DRiP and INS-splice did not co-localise with insulin, implying that SPLICE_81-95_^+^ cells are not beta cells (Fig. 2b). To assess the identity of these SPLICE_81-95_^+^ cells, human pancreatic sections were co-stained with various endocrine cell markers (insulin, glucagon and somatostatin). Staining of the SPLICE_81-95_ epitope proved restricted to delta cells as indicated by its exclusive co-localisation with somatostatin (Fig. 2c).

Since the alternatively spliced insulin isoform product shares an N-terminus with PPI, we tested whether the presence of the signal peptide contributes to post-translational processing and intracellular localisation of INS-splice. Detailed examination of pancreatic slices by high-resolution EM with quantum dot-labelled SPLICE_81-95_ antiserum demonstrated that INS-splice was localised to secretory granules of delta cells (Fig. 2d–f), which could be clearly distinguished from insulin secretory granules of beta cells by their unique ultrastructure [33]. This confirms that INS-splice is transported to delta cell granules and implies that it is secreted upon degranulation. Of note, staining of other endocrine tissues demonstrated that the presence of the INS-splice polypeptide is limited to pancreatic islets (ESM Fig. 3). Immunohistochemistry of mouse pancreas sections revealed the presence of INS-splice in delta cells, similar to humans (ESM Fig. 4).

### SPLICE_81-95_ antiserum does not cross-react with somatostatin

To validate the presence of INS-splice in delta cells and exclude cross-reactivity with somatostatin, HEK293T cells expressing *INS* were generated. Expression of *INS* in these cells led to expression of both full-length PPI mRNA and the alternatively spliced insulin mRNA variant, as observed in human islets (ESM Fig. 5a, b). Western blot analysis of lysates of these surrogate beta cells indicated that PPI is expressed, as well as an insulin isoform detected by SPLICE_81-95_ antiserum (ESM Fig. 5c). To confirm that INS-splice was detected with the SPLICE_81-95_ antiserum (and not INS-DRiP), both *INS* mRNA variants were isolated and their cDNAs cloned into separate expression plasmids. Western blot analysis of lysates of HEK293T cells transfected with the alternatively spliced insulin cDNA plasmid demonstrated that the SPLICE_81-95_ antiserum specifically detects INS-splice only, whereas cells transfected with the full-length PPI cDNA plasmid showed C-peptide expression only (ESM Fig. 5d–f).

SPLICE_81-95_ antiserum did not cross-react with recombinant somatostatin, as assessed by western blot (ESM Fig. 5g). In addition, antibody blocking assays using recombinant somatostatin did not affect detection of SPLICE_81-95_ antiserum to recombinant INS-splice, while antibody blocking with recombinant INS-splice markedly reduced INS-splice detection (ESM Fig. 5h). Similarly, blocking of SPLICE_81-95_ antiserum using the immunisation peptide reduced the mean fluorescence of the SPLICE_81-95_^+^ islet cell population compared with irrelevant peptide (ESM Fig. 6).

### Alternatively spliced *INS* RNA is expressed in beta and delta cells

**Fig. 3:**
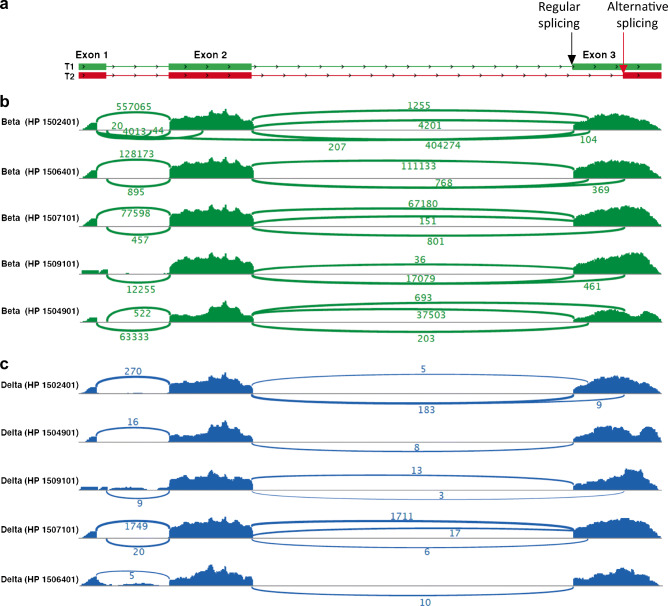
Splicing events of insulin transcripts in human beta cells and human delta cells. (**a**) Splicing pattern of PPI (T1, green) and an in silico predicted *INS* RNA splice variant (T2, red). Exons are shown as boxes and introns as lines. (**b**, **c**) Sashimi plots show splicing events of *INS* RNA for beta cells (**b**, green) and delta cells (**c**, blue). Human pancreas donor identity numbers are indicated by HP. The numbers of splicing events are shown. Splicing of PPI mRNA is shown by alignment with the regular splicing pattern from (**a**) (T1, green boxes). Alternative splicing of insulin transcripts is defined as any sequence that does not align with the regular splicing pattern as displayed in T1

To further validate the presence of spliced *INS* mRNA in delta cells, we used a publicly available single islet cell transcriptome dataset and adopted the validated cell type classification of Segerstolpe et al [1], characterised by discrete clusters of endocrine cell types (ESM Fig. 7a). All delta cells showed high expression levels of somatostatin and negligible levels of beta cell-specific transcription factor MafA (ESM Fig. 7b). We searched for supporting reads of the alternative splice junction in both beta and delta cells. Several insulin transcripts were present in beta cells and delta cells (Fig. 3). Among the alternative *INS* mRNA splice variants, the one using the cryptic splicing site within exon 3 was detected in a subset of delta cells and beta cells. Splicing of insulin transcripts was studied in beta cells and delta cells of five non-diabetic human donors. Of note, aside from the regular and alternatively spliced insulin transcripts coding for PPI and INS-splice, respectively, we report additional alternatively spliced insulin transcripts detectable in subsets of delta cells and beta cells with alternative splice acceptor sites in exon 2 and exon 3 of the *INS* RNA (Fig. 3). Furthermore, low levels of alternatively spliced *INS* mRNA were detected in the alpha, epsilon and gamma cell clusters, although mean transcripts per million (TPM) values were 2.8, 2.8 and 2.4 times lower, respectively, compared with delta cells, and 79, 77, 67 times lower, respectively, compared with beta cells (ESM Fig. 7b).

### Insulin B chain expressed in delta cells

**Fig. 4:**
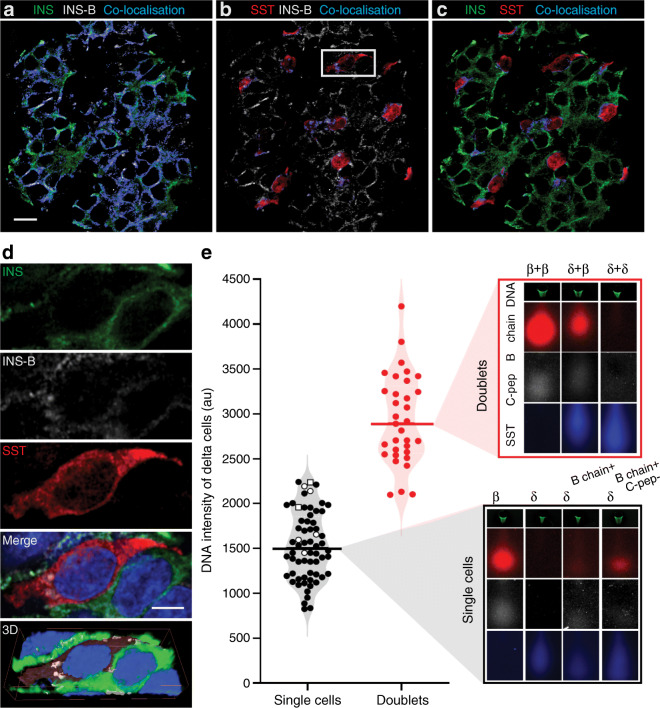
Insulin B chain expression in delta cells. (**a**–**c**) Pancreas section was stained for insulin (green), insulin B chain (white), somatostatin (red) and Hoechst. *Z*-stack images were made and used for 3D reconstruction and co-localisation analysis (Imaris). Co-localisation of insulin and insulin B chain (**a**), insulin B chain and somatostatin (**b**), and insulin and somatostatin (**c**) was determined and a co-localisation channel (blue) was created for double-positive voxels. The MCC was 0.22, 0.13 and 0.22, respectively. Scale bar, 10 μm. (**d**) Enlarged images of the grey enclosure show insulin B chain expression in a delta cell, including a 3D reconstructed image (see ESM Video). Scale bar, 5 μm. (**e**) Single-cell western blot was performed on dispersed pancreatic islet cells from two different donors and stained for DNA (green), insulin B chain (red), C-peptide (white) and somatostatin (blue). Doublets were excluded by measuring a composite of DNA intensity and hormone content of all endocrine cells. Single cells (black circles) were included for analysis on the basis of their single-cell DNA intensity and hormone content. Doublets (red circles) were excluded because of their high DNA intensity and/or high hormone content. Examples of doublets and single cells are shown. The single-cell example shows single delta cells that are positive for somatostatin and insulin B chain (δ^B chain+^, black open shapes), of which some are negative for C-peptide (δ^B chain+, C-pep−^, open squares). DNA intensity and examples of cells are shown from one out of six single-cell western blot chips. In total, 554 single delta cells were included for analysis; 54 single delta cells expressed insulin B chain besides somatostatin (9.7%), of which seven were negative for C-peptide (1.3%). Β, beta cell; δ, delta cell; INS, insulin; INS-B, insulin B chain; SST, somatostatin

Since an alternatively spliced insulin gene product containing the insulin B chain was detected in delta cells, we analysed the presence of the insulin B chain fragment in the human pancreas by immunofluorescence. Co-localisation of insulin B chain, insulin and somatostatin was determined (Fig. 4a–c). Although complete co-localisation between insulin and insulin B chain was expected, we only found 22% co-localisation, indicating that the insulin B chain staining is a gross underestimation, likely due to a low sensitivity of the antibody against insulin B chain compared with the ‘gold standard’ insulin polyclonal antibody from Dako (Fig. 4a). Importantly, some locations showed co-localisation of somatostatin and insulin B chain in the absence of insulin staining (Fig. 4b,d, grey enclosure). 3D reconstruction showed that this insulin B chain staining was indeed inside delta cells (Fig. 4d and ESM video)
ESM Video3D reconstruction of insulin B-chain expressing delta cell. Pancreas section was stained for insulin (green), insulin B-chain (white), somatostatin (red) and Hoechst (blue). 3D colocalization analysis was performed using Imaris 9.7.1 software and a video was created using Amira 2019.1 software. Insulin B-chain was shown inside the delta cell (MP4 11584 kb). Control staining with secondary antibody alone was negative (ESM Fig. 8). These data confirm insulin B chain expression in a subset of delta cells.

To validate and enumerate insulin B chain expression in delta cells, a single-cell western blot was performed on dispersed islet cells from two human donors (1000 islet equivalents each), using antibodies against somatostatin, C-peptide and insulin B chain. Only single delta cells (*n*=554) were included for analysis, and doublets were excluded by measuring a composite of their DNA intensity and hormonal content (Fig. 4e). A subset of delta cells was found that expressed insulin B chain besides somatostatin (*n*=54, 9.7%), of which some were negative for C-peptide (*n*=7, 1.3%). Detection of insulin B chain without C-peptide indicates the presence of INS-splice and excludes the presence of PPI in this subset of delta cells. These results provide evidence of the presence of *INS* products, in particular the immunogenic B chain, in a subset of delta cells.

### INS-splice activates PPI-specific CTLs

**Fig. 5:**
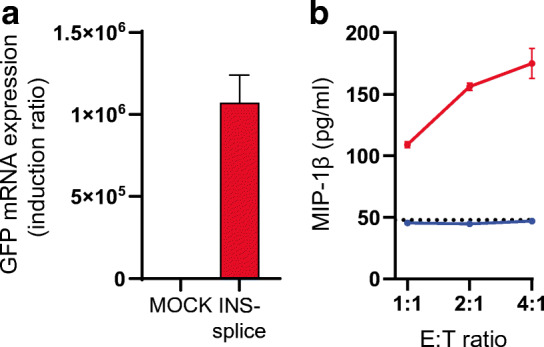
INS-splice activates PPI_15-24_-specific CTLs. (**a**) GFP mRNA expression in HEK293T cells transfected with INS-splice-IRES-GFP for 24 h. Gene expression levels are corrected for *GAPDH* used as housekeeping gene and presented as the induction ratio (mock control set to 1) (*n*=3). (**b**) MIP-1β secretion of PPI_15-24_-specific CTLs after co-culture with INS-splice-expressing HEK293T (red) or mock control (blue) cells. Effector/target ratios were 1:1, 2:1 and 4:1. The dotted line shows the basal MIP-1β secretion of PPI_15-24_-specific CTLs in the absence of target cells. Data are shown as mean ± SD, *n*=4. E, effector; T, target

INS-splice includes the complete signal peptide and B chain sequences of PPI that contain highly immunogenic epitopes targeted in individuals with type 1 diabetes [22, 24]. To investigate the immunogenicity of INS-splice, HEK293T cells were transfected with a vector encoding for INS-splice-IRES-GFP and GFP expression was validated by RT-PCR 24 h post transfection (Fig. 5a). The activation of PPI_15-24_-specific CTLs was determined by measuring their MIP-1β secretion into the supernatant fraction after co-culture with INS-splice-expressing HEK293T cells. In the absence of INS-splice expression, MIP-1β secretion was basal, while its secretion was highly upregulated in the presence of INS-splice in an effector dose-dependent manner (Fig. 5b). These data demonstrate that human cells can generate immunogenic epitopes from the INS-splice polypeptide that are processed and presented to patient-derived cytolytic T cells, suggesting that INS-splice-expressing delta cells may be targeted by autoreactive T cells in type 1 diabetes immunopathology.

### IDE cleaves INS-splice and lack of IDE expression in delta cells correlates with presence of INS-splice

**Fig. 6:**
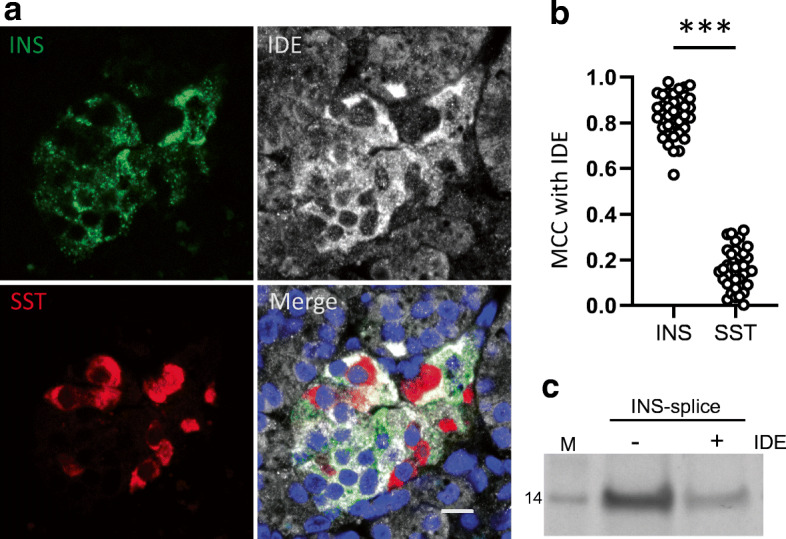
IDE is not expressed in delta cells and cleaves INS-splice. (**a**) Pancreatic IDE protein expression was determined by immunohistochemistry of human pancreas sections by staining for proinsulin (green), IDE (white) and somatostatin (red). IDE was expressed ubiquitously in the exocrine and endocrine pancreas except in delta cells. Scale bar, 10 μm. Nuclei were visualised by Hoechst staining (blue). (**b**) Co-localisation of IDE with insulin and IDE with somatostatin was quantified using MCC (QuPath). Thirty-six islets from six pancreas donors were analysed. Data are shown as mean ± SD and statistical analysis was performed using a paired two-tailed Student’s *t* test (***p<0.001). (**c**) Coomassie staining of INS-splice after IDE cleavage assay. Absence or presence of IDE is indicated by − or +, respectively. Full-length recombinant INS-splice is 14 kDa. INS, insulin; M, protein marker; SST, somatostatin

The alternatively spliced *INS* mRNA was detectable in both beta and delta cells, whereas the INS-splice protein was only detected in delta cells using SPLICE_81-95_ antiserum. To reconcile this conundrum, we hypothesised whether this INS-splice protein could be selectively degraded in some endocrine cell types but spared in others. IDE is known to capture the insulin B chain to degrade insulin [34]. Since INS-splice contains the full insulin B chain, we tested whether detection of IDE in human pancreas sections by immunohistochemistry could help explain the expression of INS-splice protein in delta cells vs beta cells. While IDE expression was confirmed ubiquitously in islets and surrounding exocrine tissue, including beta cells, it was undetectable in delta cells (Fig. 6a). Co-localisation was quantified by analysing 36 islets from six donors and the mean MCC of IDE with somatostatin (0.13) was significantly lower than that for IDE with insulin (0.82) (Fig. 6b). Next, we tested whether IDE could cleave INS-splice. Recombinant INS-splice was incubated with human IDE and visualised by Coomassie staining. INS-splice was indeed digested by IDE (Fig. 6c and ESM Fig. 9). This supports a role for IDE in the selective expression of INS-splice protein in delta cells, reconciling the discrepancy between INS-splice RNA and protein expression in beta and delta cells.

## Discussion

While insulin has been widely studied for its role in glucose homeostasis and islet autoimmunity in type 1 diabetes, little is known about alternative *INS*-derived proteins. In this study, we investigated two alternative *INS-*derived polypeptides; INS-DRiP, a defective ribosomal *INS* product; and INS-splice, an insulin splice variant that shares sequence homologies with PPI and INS-DRiP. We have previously identified INS-DRiP in type 1 diabetes immunopathology as target of cytolytic CTLs in individuals with type 1 diabetes [32]. We now report that alternative splicing of *INS* pre-mRNA resulted in a translated polypeptide with an N-terminus overlapping with PPI and a C-terminus overlapping with INS-DRiP. Similar to full-length PPI mRNA, the alternatively spliced *INS* mRNA variant was detected in both beta and delta cells by analysis of online single-cell transcriptome databases of human pancreatic islets [1], while protein detection of PPI in beta cells and INS-splice in delta cells appeared mutually exclusive. This points to cell-specific translation or protein degradation machinery in adult endocrine cells. The N-terminus of INS-DRiP containing the CTL epitope is lacking in INS-splice and can be detected in pancreatic beta cells by immunohistochemistry, supporting our previous data showing beta cell selectivity. INS-DRiP-reactive CTLs that were isolated from individuals with type 1 diabetes would therefore not be able to cross-react with INS-splice [32]. T cells reactive to INS-DRiP were present in insulitic lesions of individuals with type 1 diabetes [24]. The absence of the SPLICE_81-95_ antiserum epitope shared between INS-DRiP and INS-splice protein in beta cells suggests that INS-DRiP is rapidly targeted for protein degradation during translation in beta cells, consistent with the classic degradation process of non-stop proteins [35]. Similar to INS-DRiP, the INS-splice open reading frame lacks a stop codon, but INS-splice was detected specifically in delta cell granules using SPLICE_81-95_ antiserum. The presence of the PPI signal peptide may target the co-translational translocation of INS-splice to the ER lumen and subsequently into secretory granules, which may in turn explain why this non-stop protein was not subject to the degradation process that removes INS-DRiP from beta cells. Non-stop proteins targeted to the ER block translocon channels, which should be cleared rapidly to allow normal protein influx into the ER. Clearance of the blocked translocon channel allows the release of non-stop proteins into the ER, preventing proteasomal degradation in the cytosol [36]. We propose that IDE may be involved in the degradation and removal of INS-splice protein from beta cells. Indeed, IDE can digest INS-splice in vitro and IDE expression is ubiquitous in the pancreas except delta cells, possibly explaining why INS-splice protein is only detected in delta cells but not in beta cells despite the presence of INS-splice mRNA in both islet cell types.

Both single-cell RNA-seq and single-cell western blot have limited sensitivity in detecting rare targets [37, 38]. The low expression rate of *INS*-derived products in delta cells (i.e. alternatively spliced insulin RNA and INS-splice protein) is conceivably close to the sensitivity limit of these single-cell-based methods; this could help explain why SPLICE_81-95_ antiserum detected INS-splice in all delta cells using immunohistochemistry, while INS-splice mRNA and the insulin B chain were only detected in a delta cell subset. Alternatively, the observation that delta cells stained with SPLICE_81-95_ antiserum only partially stain with anti-insulin B chain antibody suggests that additional *INS*-splice products may be expressed in delta cells, as indicated by our transcriptome analyses as well as by detection of C-peptide traces in some delta cells containing insulin B chain. Detection of these potential additional alternatively spliced *INS* products is hampered by variation in protein length (lacking stop codon), rapid degradation of non-conventional proteins, low expression levels and limited availability of specific detection methods. Our data suggests that there are subpopulations of delta cells, as previously demonstrated in beta cells. Delta cell subsets showed heterogeneity regarding RNA expression and *INS*-derived protein expression.

The presence of the INS-splice protein in human and mouse delta cells is intriguing and may have implications for studies using the insulin promotor as a supposedly specific reporter for beta cells [39–41]. Our results imply that use of the insulin promotor activity to specifically target beta cells could lead to off-target effects in delta cells. In addition, the presence of INS-splice protein implies the activity of the insulin signal peptide and B chain in delta cells as confirmed by single-cell western blots in a subpopulation of delta cells. These peptides are major targets for islet autoimmunity [22, 23, 42–44]. INS-splice-expressing cells activated PPI-specific CTLs, validating the immunogenicity of the INS-splice peptide. Hence, a subset of delta cells may produce and present diabetogenic epitopes in HLA, making them vulnerable to attack by diabetogenic T cells. Delta cells have not yet been thoroughly investigated for their involvement in islet autoimmunity in type 1 diabetes. While impaired delta cell function has been reported [45], data on delta cell destruction by autoreactive CTLs are still lacking. INS-splice shares sequence homology with a previously described INS-IGF2 protein [46] and a 74-amino-acid proinsulin protein [47], although their C-termini differ. Since all three isoforms retained the insulin signal peptide and B chain, the intracellular distribution and function may overlap.

The relevance of *INS* mRNA expression in non-beta endocrine cells remains unclear. Delta cells have an important role in beta cell development during organogenesis [48, 49]. Human beta cells have also been shown to change identity via de- and transdifferentiation [11]. Alternative splicing is involved in maintaining lineage differentiation and tissue identity as well as maintenance of cell pluripotency and is influenced by the microenvironment [17]. It remains unknown whether *INS* promoter activity in non-beta cell endocrine cells is a remnant of their common progenitor cell or contributes to maintaining endocrine cell plasticity in adolescence. While the function of INS-splice protein is still enigmatic, its presence in secretory granules implies that INS-splice is co-secreted with somatostatin during exocytosis and may have paracrine or endocrine function in the developmental destiny of human islet cells.

## Supplementary information

ESM (PDF 1.99 mb)

ESM QuPath Colocalisation Script (DOCX 17.7 kb)

ESM Video 3D reconstruction of insulin B-chain expressing delta cell. Pancreas section was stained for insulin (green), insulin B-chain (white), somatostatin (red) and Hoechst (blue). 3D colocalization analysis was performed using Imaris 9.7.1 software and a video was created using Amira 2019.1 software. Insulin B-chain was shown inside the delta cell (MP4 11.3 MB)

## Data Availability

The full EM dataset is available via www.nanotomy.org (for review: http://www.nanotomy.org/OA/Tienhoven2021SUB/6126-368/). Single-cell RNA-seq data was made available by Segerstolpe et al [1] and can be found at https://sandberglab.se/pancreas. The RNA and protein sequence of INS-splice was uploaded to GenBank (BankIt2546444 INS-splice OM489474).

## Abbreviations

CTL: Cytotoxic T lymphocyte
EM: Electron microscopy
ER: Endoplasmic reticulum
IDE: Insulin-degrading enzyme
INS-DRiP: Insulin defective ribosomal product
INS-splice: Alternatively spliced insulin product
MCC: Manders co-localisation coefficient
PPI: Preproinsulin
MIP-1β: Macrophage inflammatory protein-1 β
