# Supplementary material for: Presence of immunogenic alternatively spliced insulin gene product in human pancreatic delta cells
Source: Diabetologia. 2023 Mar 8;66(5):884–96. doi: 10.1007/s00125-023-05882-y (PMC10036285; doi:10.1007/s00125-023-05882-y)
Supplement: Supplementary file 2 — (PDF 1.99 mb) [file 125_2023_5882_MOESM1_ESM.pdf]

Electronic Supplementary Material (ESM), van Tienhoven et al.

ESM methods

Human islets

Pancreatic islets were obtained from human cadaveric donor Pancreases. Islets that could not be used for clinical transplantation were made available for research for which consent was present. The reported investigations were carried out in accordance with the principles of the declaration of Helsinki as revised in 2008. Human islets were isolated as previously described [1]. For RNA analysis and flow cytometry, islets were isolated in the GMP-facility of LUMC and maintained in ultra-low attachment plates (Corning, NY 14831, United States) in low glucose DMEM supplemented with 10% FBS, 100 units/ml Penicillin and 100 µg/ml streptomycin (Gibco-BRL, United States). For single cell western blot, islets were isolated in the GMP-facility of City of Hope and cultured in Connaught Medical Research Laboratories (CMRL)-1066 media supplemented with 0.5% human serum albumin (Baxter Healthcare Corporation, United States) and 0.1 µg/ml insulin-like growth factor-1 (Cell Sciences, United States).

DNA constructs and transfection

To generate insulin expressing vectors, human genomic DNA was isolated from HEK cells. The human *INS* gene was cloned by PCR using Phusion High-fidelity DNA Polymerase with the following primers: INS Fw 5'-AGCCCTCCAGGACAGGC-3' and INS Rv 5'-TTTTGCTGGTTCAAGGGCTTTATT-3'. The PCR fragment was cloned into pJet1.2/blunt vector and subcloned into pRRL-CMV vector to generate a pLV-CMV-INS/full expressing the human *INS* gene. The same PCR primers were used on oligo(dT) primed cDNA synthesized from purified human islet RNA. The two PCR

fragments obtained were cloned into pJET1.2/blunt and subsequently cloned into pRRL-CMV-IRES GFP to generate a pLV-CMV-INS/wt-bc-GFP and pLV-CMV-INS/splice-bc-GFP. The constructs were verified by Sanger sequencing using primer located into the CMV promoter.

HEK293T cells were transfected in suspension using polyethylenimine (PEI). A transfection mixture for one well of a 6-well plate consisted of 2 µg plasmid DNA and 6 µg PEI (pH 7.4) supplemented with Opti-MEM I reduced serum medium (Gibco, United States) to a final volume of 200 µl. Mixes were thoroughly mixed and pre-incubated for 10 min at RT before adding to the cell suspension, 48h post transfection cells were harvested for further analysis.

Western blotting

Cells were lysed using Tropix lysis mix (Applied Biosystems, United States) and protein content was determined by Bradford reagent assay (Biorad, Veenendaal, The Netherlands). For analysis, 50 µg protein sample was used. Samples were boiled in sample buffer (10% glycerol, 2% SDS, 50 mM Tris-HCl pH 6.8, 0.1% Bromophenol blue and 1% β-mercaptoethanol) for 5 min before loading onto a 15% SDS-polyacrylamide gel. Proteins were subsequently transferred onto 0.2 µm pore size Immobilon-P (Merk Millipore, United States) and visualized by standard protocols with anti-insulin (1:1000, H-86 sc-9168), anti-C-peptide (1:1000, CBL94 Millipore, United States), anti-actin (1:5000, C4 MAB1501 Merck Millipore, United States), anti-GFP (1:2000, A11122 Invitrogen, United States), anti-SPLICE$_{81\text{-}95}$ (1:1000) and anti-somatostatin (1:1000, 13-2366 Emelca Bioscience, Germany). For antibody blocking assays, the primary antibody was blocked with 5 µg recombinant polypeptide

overnight at 4C prior addition to the membranes. After this standard western blot protocols were followed.

Flow cytometry

Prior flow cytometric analysis of human pancreatic islets, single cells were achieved by dispersion using trypsin and filtration. Thereafter, the islets cells were fixed and permeabilized with 4% PFA, 0.1% saponin in PBS for 30 min at 4ºC. Islet cells were subsequently stained with anti-$SPLICE_{81-95}$ as primary antibody (1:500) and Alexa-568-conjugated secondary antibodies against rabbit (Life technologies 1:500, United States). Antibodies were diluted in 0.1% saponin, 1% BSA in PBS and incubated for 1h cold on ice. Islet cell populations were analysed and sorted using a FACS Aria II (BD biosciences, United States).

Enzyme-linked immunosorbent assay

Nunc Maxisorp 96 well plates (Sigma-Aldrich, United States) were coated with 1 µg/well of the desired recombinant polypeptide diluted in PBS to reach a final volume of 100 µl/well and incubated overnight at 4C. After coating, the wells were blocked with 200 µl/well 2% BSA in PBS for 1h at RT, followed by 2h incubation with primary antibodies against $DRiP_{1-13}$ (1:1000), $SPLICE_{81-95}$ (1:1000) or C-peptide (1:2500). And subsequently visualized 2h with HRP-conjugated secondary antibody anti-rabbit and anti-mouse (Santa Cruz, United States). Antibodies were diluted in 1% BSA in PBS to reach a final volume of 100 µl/well. For detection, 100 µl/well of freshly prepared substrate, 0.4 mg/ml O-phenylenediamine (P9029, Sigma, United States) in 0.05M phosphate-citrate buffer pH 5.0, was used. After 15 min incubation the absorbance at 450 nm was measured. After each step, wells were aspirated and extensive washed

using 200 µl/well Wash buffer (0.05% Tween-20 in PBS). This was repeated for a total of 5 washes with 1 min soaking to increase the effectiveness of the washes.

Electron microscopy (EM)

EM islet datasets were created from nPOD donors. Additional donor details can be obtained through the JDRF nPOD online pathology database. Tissues were recovered following informed research consent from next of kin in the United States and shipped to the nPOD program at the University of Florida for processing as previously described [2]. All experiments were conducted under the approval of the University of Florida Institutional Review Board and the current study fulfills all requirements for tests as approved by the medical ethical review board of the University Medical Center Groningen.

Pancreas samples were fixed in cold, freshly prepared 2% paraformaldehyde-1% glutaraldehyde for 48h followed by transfer to PBS for storage at 4°C before shipment to the Netherlands. Tissue vibratome sections (~50 µm; Microm HM 650V) were post-fixed in osmium tetroxide/ potassium ferrocyanide, followed by dehydration and flat-embedding as previously reported. Next, regions with islets were selected from toluidine stained 1 µm sections using light microscopy. Subsequent ultrathin (80 nm) sections were cut (UC7 ultramicrotome, Leica Microsystems, Vienna, Austria) and placed on formvar coated copper grids (Electron Microscopy Sciences, Hatfield, Pennsylvania). Finally, sections were contrasted with uranyl acetate as previously described [3].

Post-embedding immunolabeling on Epon with gold or quantum dots was carried out as described before [3], using SPLICE81-95 antiserum. Data were acquired on a Supra 55 scanning EM (SEM; Zeiss, Oberkochen, Germany) using a scanning

transmission EM (STEM) detector at 28kV with 2.5 nm pixel size with an external scan generator ATLAS 5 (Fibics, Ottawa, Canada) as previously described [3]. Beta cells and delta cells could be identified using established morphological characteristics of their secretory granules. Delta cell granules were larger and less dense compared to beta cell granules, and lacked the characteristic clear peripheral mantle of beta cell granules [3]. Stitched data are accessible at http://www.nanotomy.org/OA/Tienhoven2021SUB/6126-368/.

Immunohistochemistry and microscopy

Paraffin embedded tissues were cut into 4 μm sections. Tissues were deparaffinized in xylene and rehydrated in a series of ethanol decreasing in concentration. Prior antibody incubation antigen retrieval was performed by autoclavation in citrate buffer (pH 6.0) or tris buffer (pH 9). Sections were blocked for 1h with 2% normal donkey serum in PBS. Primary and secondary antibodies diluted in PBS/1%BSA were incubated subsequently for 1h at RT with additional washing steps in between. Primary antibodies were used against insulin (1:300, A0564, Dako, United States), C-peptide (1:500, CBL94 Millipore, United States), glucagon (1:200, ab10988 Abcam, United States), somatostatin (1:500, 13-2366 Emelca Bioscience, Germany), $DRiP_{1-13}$ (1:500, custom-made) and $SPLICE_{81-95}$ (1:500, custom-made), insulin B-chain (1:250, ab133281, Abcam, United States), insulin degrading enzyme (1:100, ab32216, Abcam, United States) and proinsulin (1:100, GS-9A8; kindly provided by Dr. O. Madsen). The appropriate Alexa-conjugated antibodies were used (anti-mouse, anti-guinea pig, anti-rat, anti-rabbit or anti-sheep). Nuclei were stained with Vectashield with DAPI (Vector laboratories #H-1500, United States) or Hoechst (33342, BD Biosciences, United States). Immunofluorescence was detected with a Leica SP8

confocal microscope or Zeiss LSM880 confocal microscope. The 3D colocalization analysis was performed using Imaris 9.7.1 software and 3D images and videos were created using Amira 2019.1 software. Colocalization between IDE and insulin or somatostatin was quantified using QuPath 0.2.3; the script used for this analysis is available in the ESM. Manders Colocalization Coefficients were used to investigate whether IDE was present more frequently within somatostatin or insulin positive areas.

CTL activation assay

HEK293T cells were transfected with a bicistronic vector encoding the alternatively spiced insulin mRNA together with a GFP reporter under the control of a CMV promoter. Transfection was performed using Lipofectamine 2000 according to the manufacturer's protocol (Thermo Fisher Scientific, United States). GFP expression was validated by RT-PCR using GFP-specific primers. 24h post-transfection, target cells were cocultured with CTLs directed against the PPI signal peptide PPI_{15-24} at Effector:Target ratios of 1:1, 2:1 and 4:1. Cocultures were incubated at 37°C for 3h in IMDM supplemented with 10% human albumin and 25U/ml IL2 (Novartis, United States). The supernatant was used for detection of MIP-1β production by the CTLs, using the MIP-1β ELISA kit (# 88-7034-22; Thermo Fisher Scientific, Unites States), according to the manufacturer's protocol.

References

[1] Ricordi C, Lacy PE, Finke EH, Olack BJ, Scharp DW (1988) Automated method for isolation of human pancreatic islets. Diabetes 37(4): 413-420. 10.2337/diab.37.4.413

[2] Campbell-Thompson M, Wasserfall C, Kaddis J, et al. (2012) Network for Pancreatic Organ Donors with Diabetes (nPOD): developing a tissue biobank for type 1 diabetes. Diabetes Metab Res Rev 28(7): 608-617. 10.1002/dmrr.2316

[3] de Boer P, Pirozzi NM, Wolters AHG, et al. (2020) Large-scale electron microscopy database for human type 1 diabetes. Nat Commun 11(1): 2475. 10.1038/s41467-020-16287-5

ESM table 1: Human islet checklist

Checklist for reporting human islet preparations used in research (van Tienhoven et al.) Adapted from Hart NJ, Powers AC (2018) Progress, challenges, and suggestions for using human islets to understand islet biology and human diabetes. Diabetologia https://doi.org/10.1007/s00125-018-4772-2.

**Islet preparation**	**1**	**2**	**3**	**4**	**5**	**6**	**7**	**8[a]**
**MANDATORY INFORMATION**								
Unique identifier	X138	PANC548	PANC550	PANC551	Hu1170	Hu1185		
Donor age (years)	23	50	45	57	20	18		
Donor sex (M/F)	M	M	M	F	M	M		
Donor BMI (kg/m^2)	20	24	31	31	36	39.9		
Donor HbA_{1c} or other measure of blood glucose control	10 mM (last measurement at ICU)	9.2 mM (last measurement at ICU)	9.2 mM (last measurement at ICU)	6.4 mM (last measurement at ICU)	4.6	5.1		
Origin/source of islets[b]	LUMC	LUMC	LUMC	LUMC	IIDP/COH	IIDP/COH		
Islet isolation centre	LUMC	LUMC	LUMC	LUMC	COH	COH		
Donor history of diabetes? Please select yes/no from drop down list	No	No	No	No	No	No		
**If Yes, complete the next two lines if this information is available**								
Diabetes duration (years)								
Glucose-lowering therapy at time of death[c]								

RECOMMENDED INFORMATION								
Donor cause of death					Head Trauma /MVA	Head Trauma /Non-MVA		
Warm ischaemia time (h)					0	0		
Cold ischaemia time (h)					7.58	11.28		
Estimated purity (%)					95	73		
Estimated viability (%)					99	93		
Total culture time (h)[d]								
Glucose-stimulated insulin secretion or other functional measurement[e]								
Handpicked to purity? Please select yes/no from drop down list								
Additional notes								

[a]If you have used more than eight islet preparations, please complete additional forms as necessary
[b]For example, IIDP, ECIT, Alberta IsletCore
[c]Please specify the therapy/therapies
[d]Time of islet culture at the isolation centre, during shipment and at the receiving laboratory
[e]Please specify the test and the results

ESM Figure 1

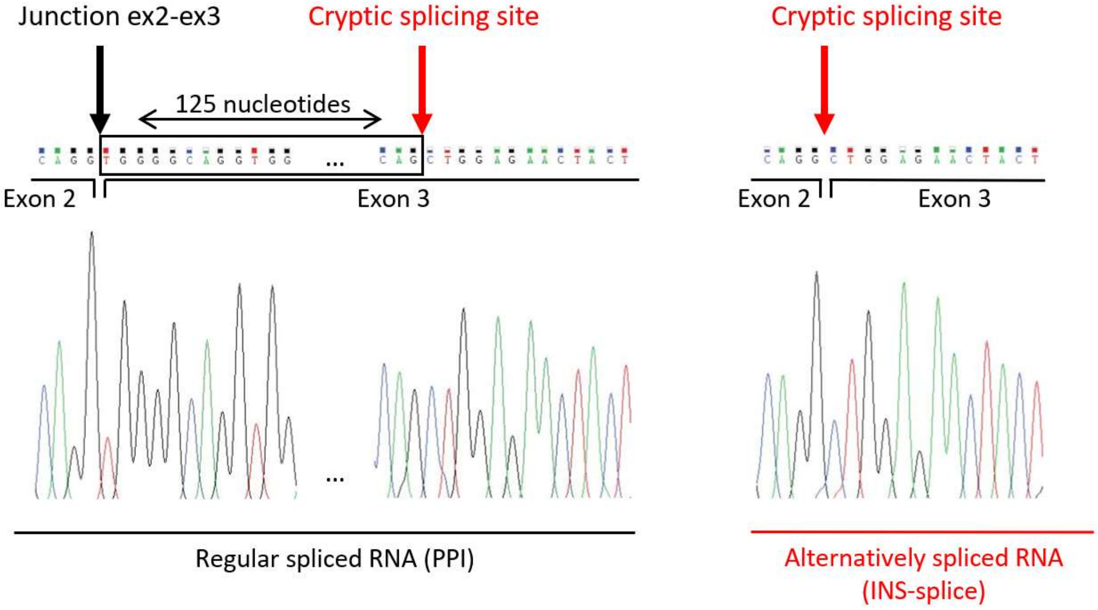

ESM figure 1: Nucleotide sequencing of splicing sites in insulin cDNA. DNA sequence analysis of the retrieved PCR fragments (from figure 1) spanning the splicing region between the 5' donor site in exon 2 and the cryptic 3' acceptor site in exon 3 of the insulin RNA. Regular splicing leads to RNA that codes for PPI (left) and alternative splicing using the cryptic splice acceptor site in exon 3 leads to RNA that codes for INS-splice (right). The regular junction between exon 2 and exon 3 (black arrow) and cryptic splicing acceptor site in exon 3 (red arrows) in the cDNA are indicated. Alternative splicing removes the first 123 nucleotides of exon 3.

ESM Figure 2

a

Recombinant PPI

1 MSYYHHHHHH LESTSLYKKA GFTMALWMRL LPLLALLALW GPDPAAAFVN QHLCGSHLVE ALYLVCGERG FFYTPKTRRE

81 AEDLQVGQVE LGGGPGAGSL QPLALEGSLQ KRGIVEQCCT SICSLYQLEN YCN*

Recombinant INS-DRiP

1 MSYYHHHHHH LESTSLYKKA GFTMLYQHLL PLPAGELLQL DAARRQPPTR RLLHRERWNK ALEPAK*

$DRiP_{1-13}$ $SPLICE_{81-95}$

Recombinant INS-splice

1 MSYYHHHHHH LESTSLYKKA GFTMALWMRL LPLLALLALW GPDPAAAFVN QHLCGSHLVE ALYLVCGERG FFYTPKTRRE

81 AEDLQAGELL QLDAARRQPP TRRLLHRERW NKALEPAK*

$SPLICE_{81-95}$

Recombinant proSST

1 MSYYHHHHHH LESTSLYKKA GFTMAPSDPR LRQFLQKSLA AAAGKQELAK YFLAELLSEP NQTENDALEP EDLSQAAEQD

81 EMRLELQRSA NSNPAMAPRE RKAGCKNFFW KTFTSC*

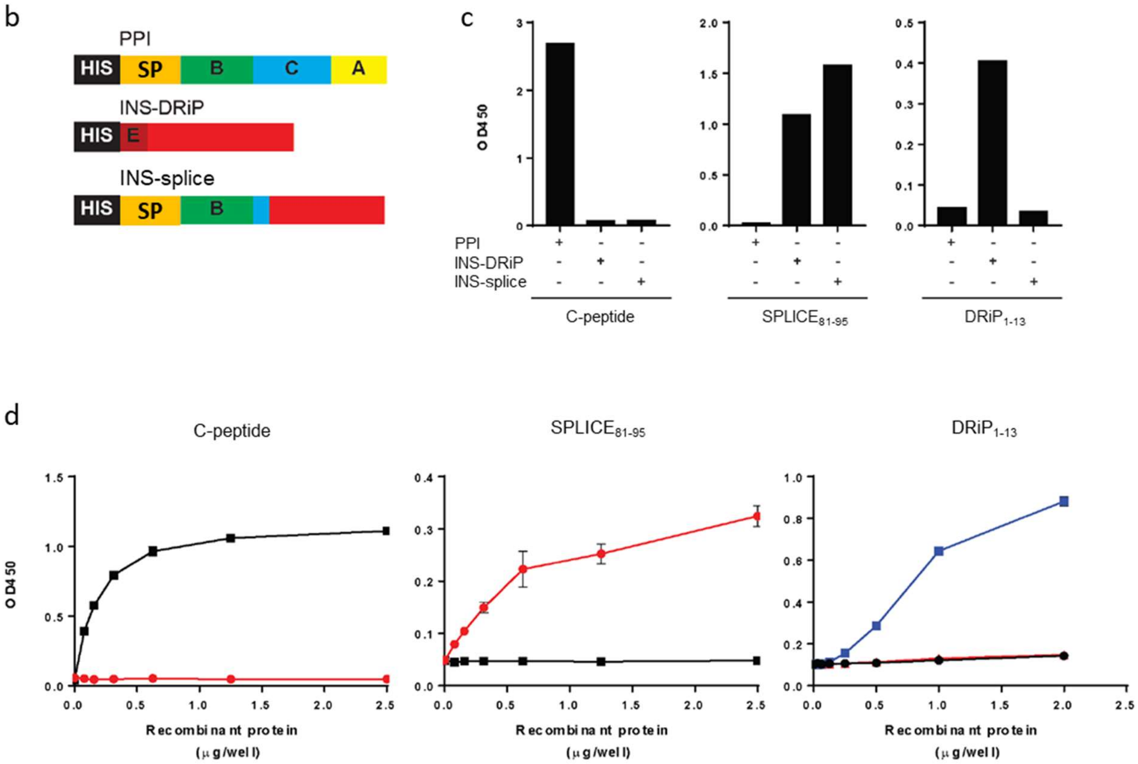

ESM figure 2: Validation of $DRiP_{1-13}$ and $SPLICE_{81-95}$ antisera.

(a) Amino acid sequence of the recombinant polypeptides. Red line indicates the location of the immunization peptides used for antiserum development. (b) Schematic representation of the recombinant polypeptides, with His-tag (black box), signal peptide (SP, orange), B-chain (B, green), C-peptide (C, blue), A-chain (A, Yellow). The frame shift of INS-DRiP and INS-splice (red) are indicated separately. Corresponding amino acid sequences are indicated with corresponding colors, letters indicate the presence of the complete chain. The E indicates the INS-DRiP-specific CD8 T-cell epitope. (c) Validation of serum specificity in an indirect ELISA assay to recombinant PPI, INS-DRiP and INS-splice using C-peptide antibody (left panel), $SPLICE_{81-95}$ antiserum (middle panel) and $DRiP_{1-13}$ antiserum (right panel). (d) Validation of antiserum response in an indirect ELISA assay to serial dilutions of recombinant PPI (black curves), INS-splice (red curves) and INS-DRiP (blue curve) using C-peptide antibody (left panel), $SPLICE_{81-95}$ antiserum (middle panel) and $DRiP_{1-13}$ antiserum (right panel).

ESM Figure 3

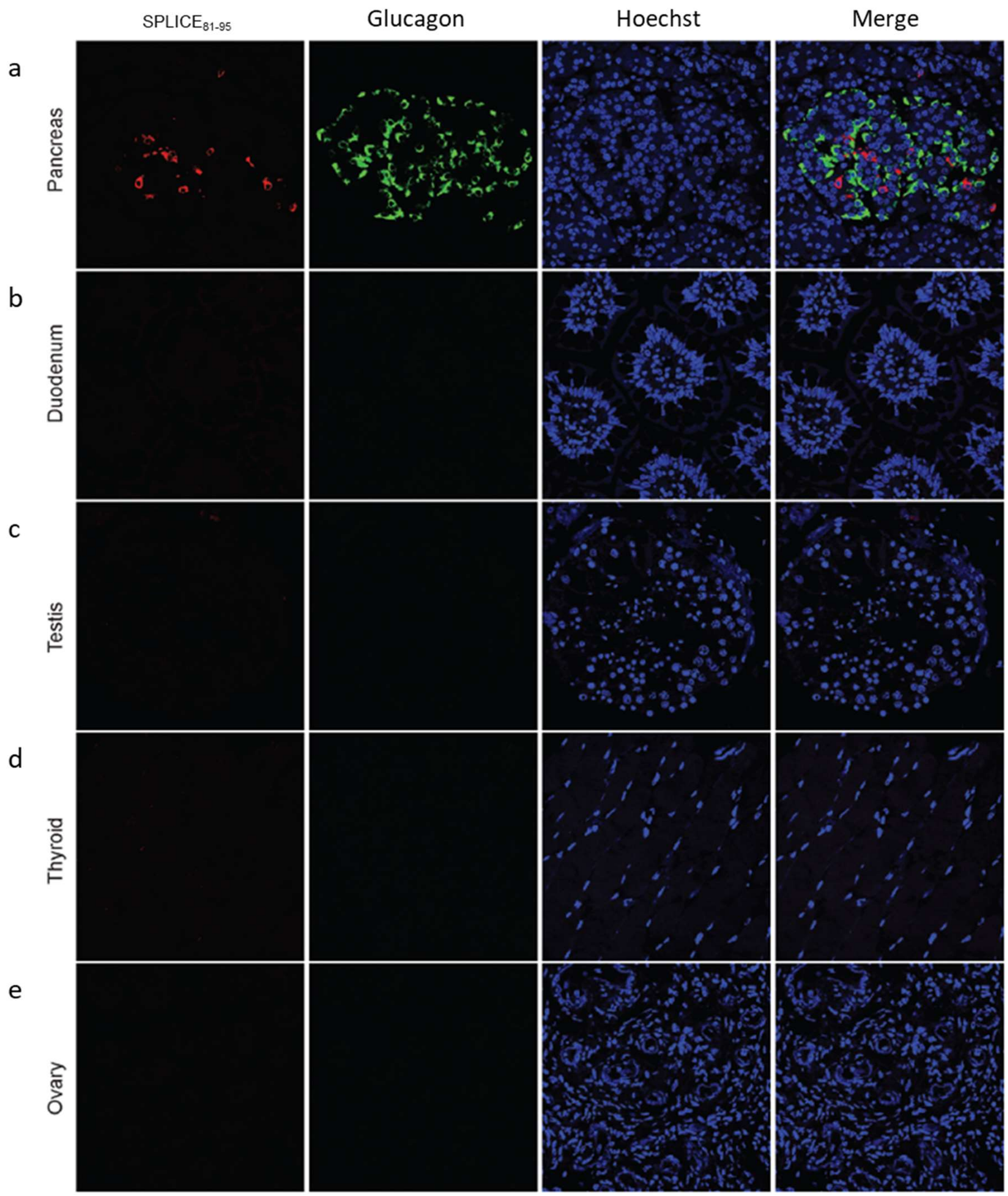

ESM figure 3: INS-splice is exclusively detected in pancreatic islets. Immunohistochemistry of a variety of human endocrine tissue for INS-splice (red) and glucagon (green). (a) Pancreas, (b) duodenum, (c) testis, (d) thyroid and (e) ovary. Nuclei were visualized by Hoechst staining (blue).

ESM Figure 4

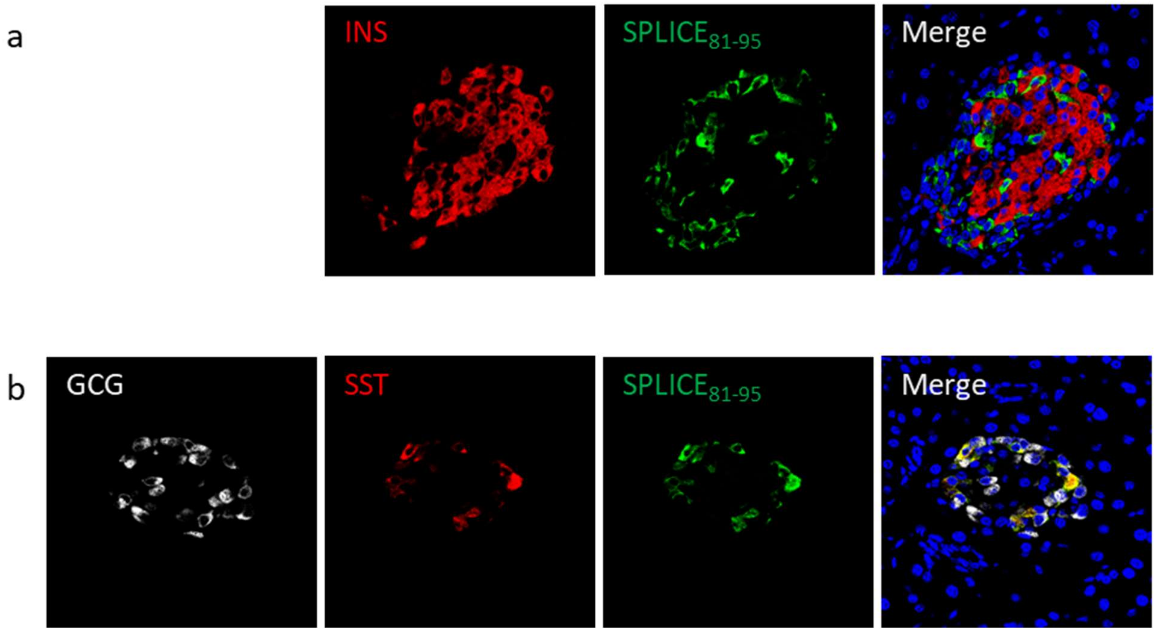

ESM figure 4: SPLICE$_{81\text{-}95}$ antiserum stained mouse delta cells. Expression of INS-splice was determined by immunohistochemistry of mouse pancreatic tissue (C57BL6). SPLICE81-95 (green) was stained in combination with insulin (red, a) and in combination with glucagon (white) and somatostatin (red, B). INS-splice was expressed in delta cells as seen by colocalization with somatostatin (b). Primary antibodies were used against insulin (ab195956 Abcam), SPLICE$_{81\text{-}95}$ (custom-made), somatostatin (Mab354 Millipore) and glucagon (ab10988 Abcam). Nuclei were visualized by Hoechst staining (blue).

ESM Figure 5

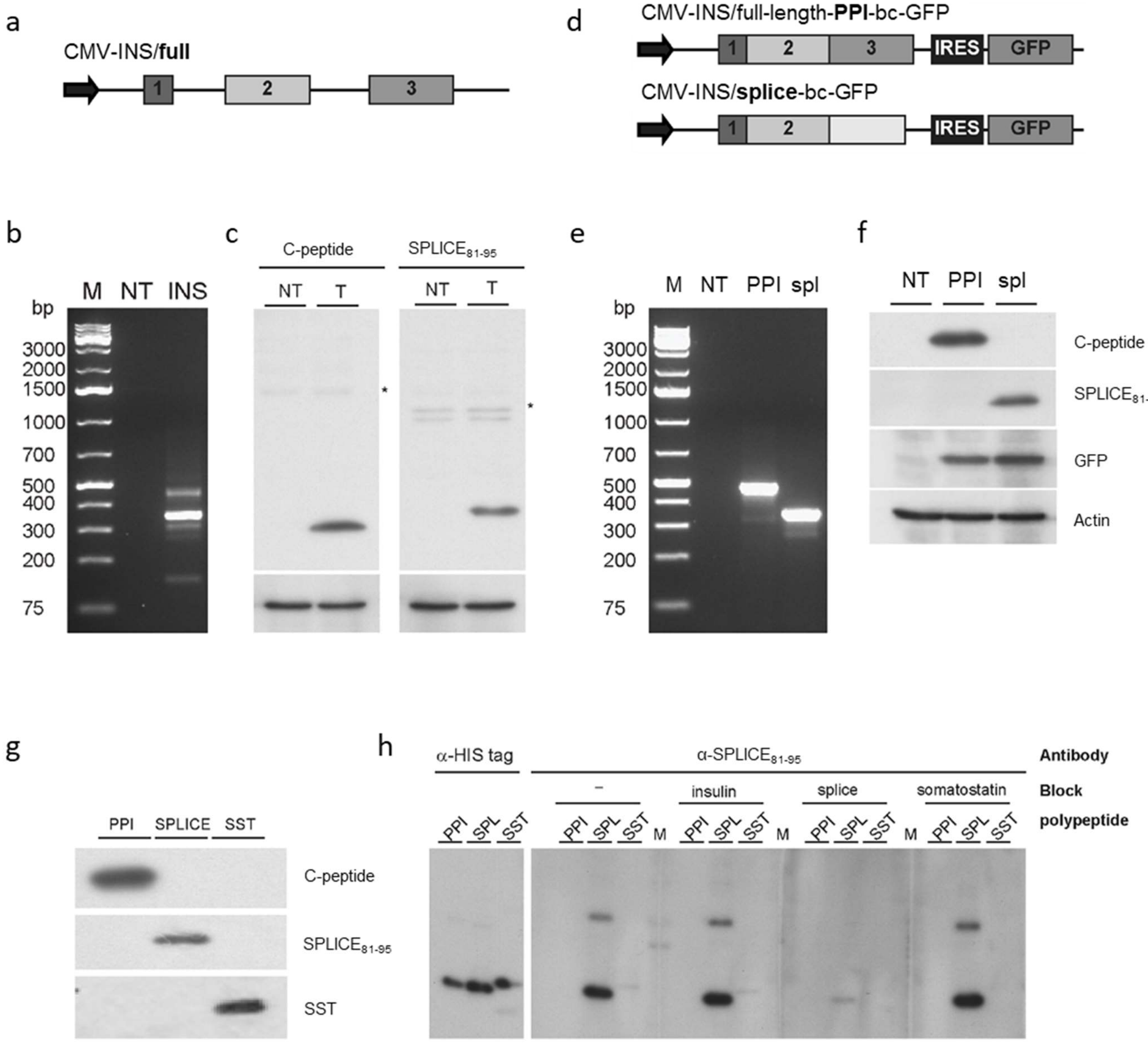

ESM figure 5: INS-splice is transcribed from *INS* and translated in HEK293T cells. (a) Schematic representation of the plasmid CMV-INS, encoding all exons (annotated by 1-3) and introns (black line) of insulin driven by a CMV promotor. (b) Analysis of insulin splicing in HEK293T cells after transfection with CMV-INS (INS) by PCR using the same primer set as described in figure 1. The DNA marker is indicated

by the M, NT= non-transfected, T=transfected. (c) Western blot analysis of HEK293T cell lysates transfected with CMV-insulin (T) and non-transfected cells (NT). Lysates were made 48h post transfection and analyzed with anti-C-peptide (left panel), $SPLICE_{81-95}$ antiserum (right panel) and anti-actin (bottom panels). Unspecific background bands (*). (d) CMV-INS/full-length-PPI-bc-GFP, encoding the full-length PPI mRNA of normally spliced insulin, and CMV-INS/splice-bc-GFP, encoding the alternatively spliced insulin mRNA. (e) PCR analysis of mRNA from HEK293T cells transfected with CMV-INS/full-length-PPI-bc-GFP (PPI) or CMV-INS/splice-bc-GFP (spl) or non-transfected cells (NT). (f) Western blot analysis of HEK293T cell lysates transfected with CMV-INS/full-length-PPI-bc-GFP (PPI) or CMV-INS/splice-bc-GFP (spl) or non-transfected (NT). Lysates were made 48h post transfection and analysed with anti-C-peptide (first panel), $SPLICE_{81-95}$ antiserum (second panel), anti-GFP (third panel) and anti-actin (fourth panel). (g) Western blot analysis of recombinant PPI, recombinant INS-splice (SPLICE) and recombinant somatostatin (SST) with C-peptide (upper panel), $SPLICE_{81-95}$ serum (middle panel) and somatostatin (lower panel). (h) Western blot analysis of recombinant polypeptides with $SPLICE_{81-95}$ antiserum that has previously been blocked overnight with recombinant PPI, INS-splice or somatostatin. 15% SDS-polyacrylamide gels were loaded with 50 ng recombinant polypeptide. Antibody blockings were performed with 5 µg recombinant polypeptide. All membranes were developed simultaneously on a single film to exclude differences in band intensity due to differences in exposure time. The M indicate the position of the protein marker were membranes were cut after blotting and put together prior development. Loading of recombinant polypeptides was demonstrated by the presence of the HIS-tag (left panel).

ESM Figure 6

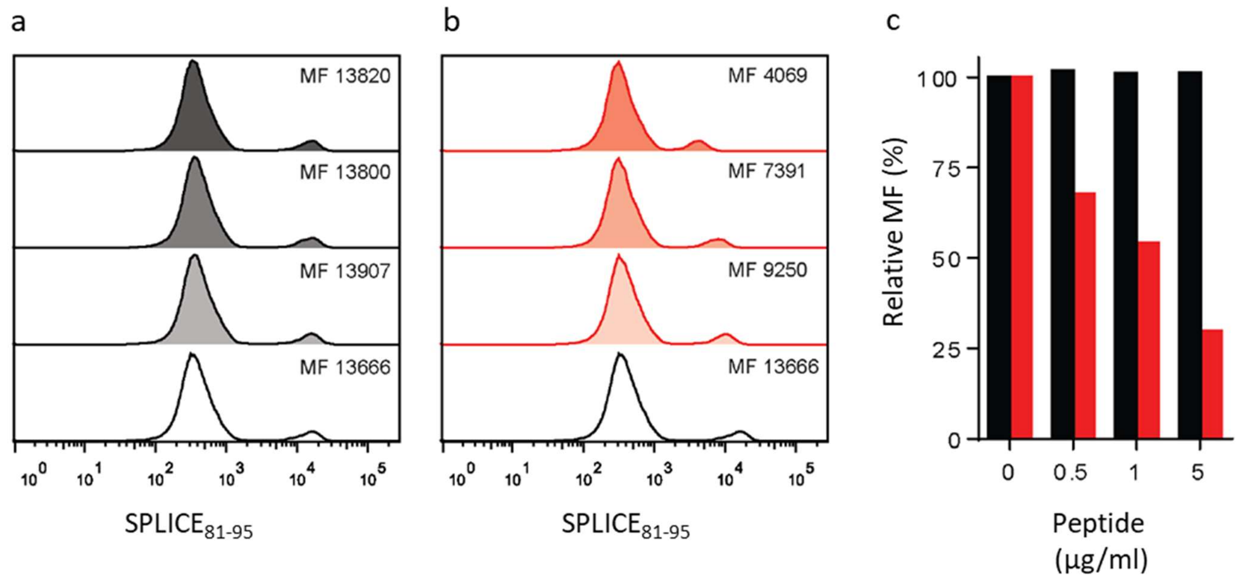

ESM figure 6: Flow cytometric analysis of human pancreatic SPLICE$_{81-95}$$^{+}$ islet cells. Flow cytometric analysis of dispersed human pancreatic islet cells stained with SPLICE$_{81-95}$ antiserum after antibody blocking with an increasing amount of irrelevant peptide (PPI$_{15-24}$) (a) and the immunization peptide (b). (c) Mean fluorescence (MF) of the SPLICE$_{81-95}$$^{+}$ cell population is depicted, relative to the unblocked MF (0 µg/ml peptide). Black and red bars represent blocking with irrelevant peptide or immunization peptide, respectively.

ESM Figure 7

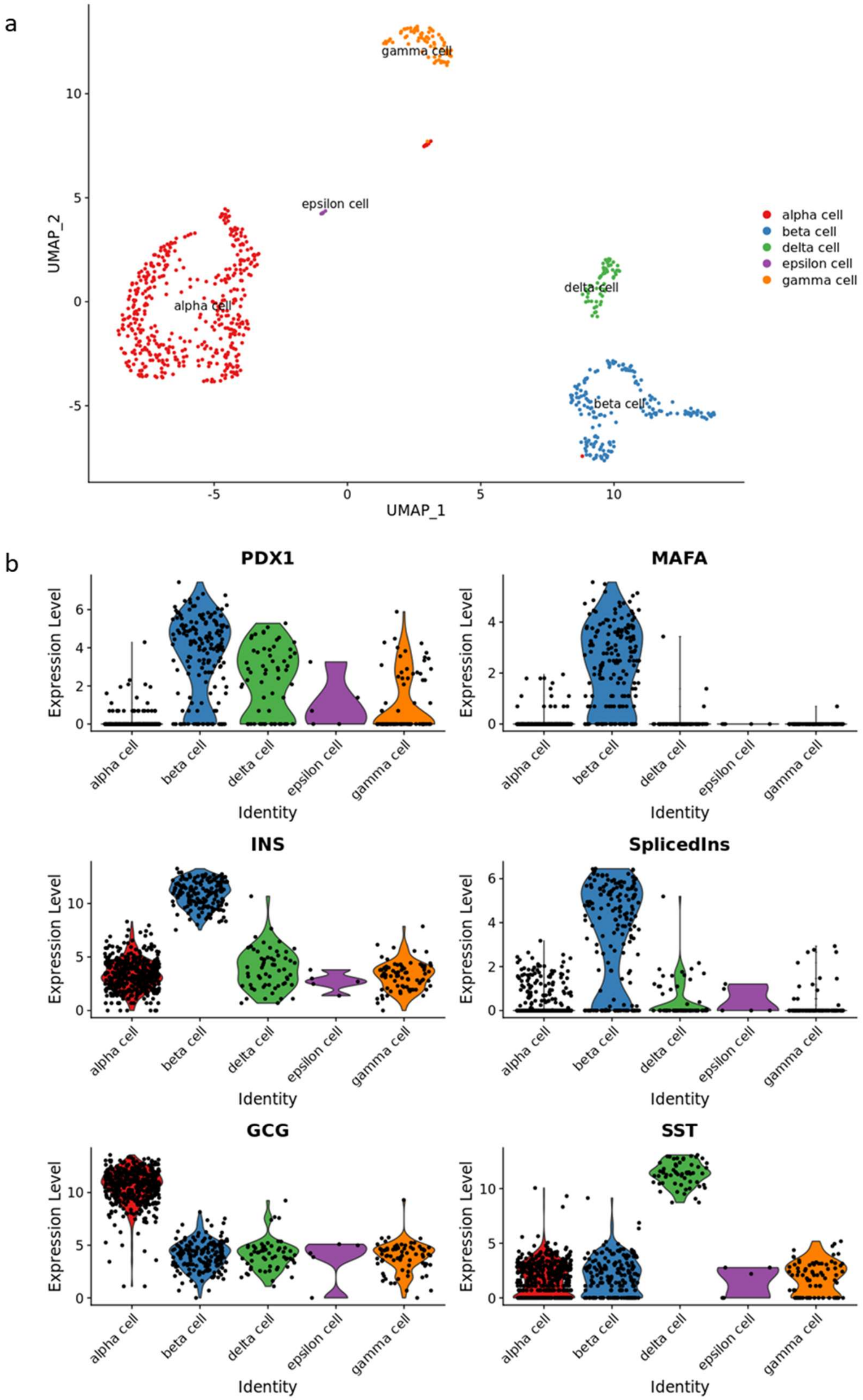
a
gamma cell
epsilon cell
alpha cell
delta cell
beta cell
UMAP_2
UMAP_1
alpha cell
beta cell
delta cell
epsilon cell
gamma cell
b
PDX1
MAFA
INS
SplicedIns
GCG
SST
Expression Level
Identity
alpha cell
beta cell
delta cell
epsilon cell
gamma cell

ESM figure 7: Single cell transcriptome analysis of pancreatic endocrine cells. (a) UMAP analysis of single cell RNA sequencing data from Segerstolpe et al. shows discreet endocrine cell subsets. (b) Expression levels of PDX1, MAFA, INS, alternatively spliced insulin (SplicedIns), glucagon and somatostatin are shown for every endocrine cell subset (alpha, beta, delta, epsilon and gamma). All delta cells show high expression of somatostatin. Alternatively spliced insulin RNA was detected in all endocrine subsets and highest levels were detected in beta and delta cells.

ESM Figure 8

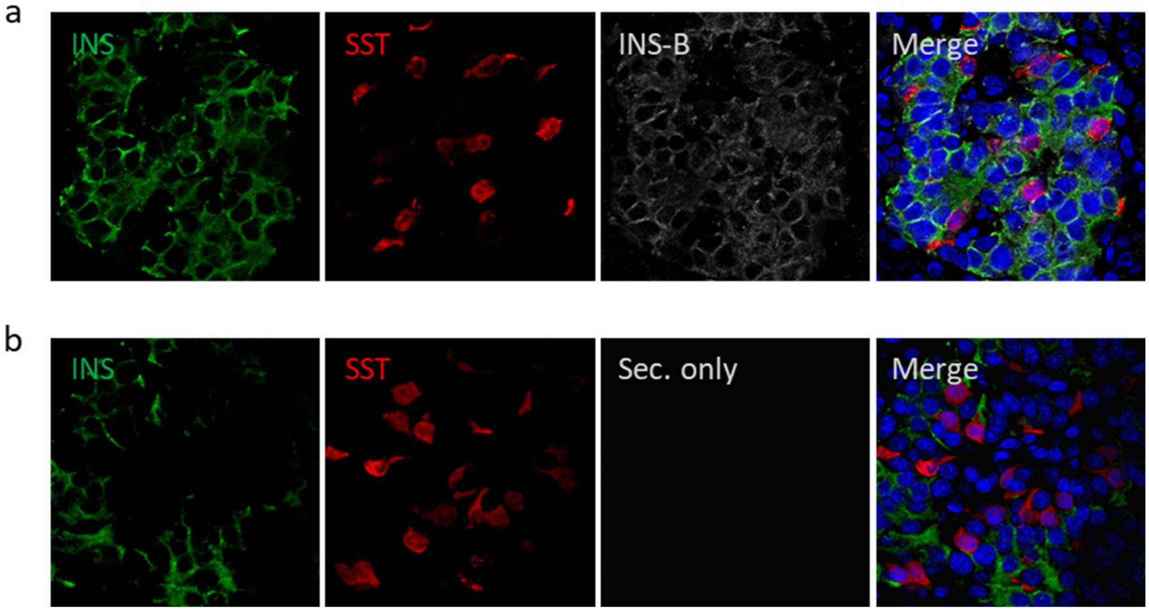

ESM figure 8: Validation of insulin B-chain antibody specificity on pancreatic tissue. Signal specificity was confirmed by comparing fully stained pancreatic tissue (a) and a similar control stain without primary insulin B-chain antibody. The secondary antibody for insulin B-chain (donkey anti-rabbit-AF647) was included to determine the background signal (b). Staining is shown for insulin (green), somatostatin (red), insulin B-chain (white) and Hoechst (blue).

ESM Figure 9

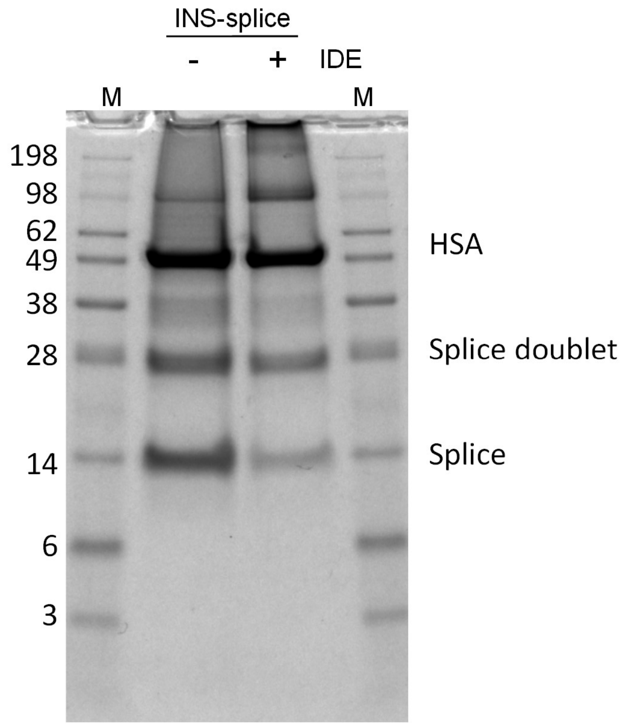

ESM figure 9: IDE cleaves INS-splice. Whole gel of Coomassie staining of INS-splice after IDE cleavage assay. Absence or presence of IDE is indicated with - or +, respectively. Full-length recombinant INS-splice is 14kDa. Protein marker is indicated by the M, numbers indicate protein size in kDa. Human Serum Albumin (HSA) was added as protein stabilizer. A doublet of INS-splice was observed at 28kDa.

ESM video legend

ESM video 3D reconstruction of insulin B-chain expressing delta cell. Pancreas section was stained for insulin (green), insulin B-chain (white), somatostatin (red) and Hoechst (blue). 3D colocalization analysis was performed using Imaris 9.7.1 software and a video was created using Amira 2019.1 software. Insulin B-chain was shown inside the delta cell
