# Supplementary material for: Presence of immunogenic alternatively spliced insulin gene product in human pancreatic delta cells
Source: Diabetologia. 2023 Mar 8;66(5):884–96. doi: 10.1007/s00125-023-05882-y (PMC10036285; doi:10.1007/s00125-023-05882-y)
Supplement: Supplementary file 3 — (DOCX 17.7 kb) [file 125_2023_5882_MOESM2_ESM.docx]

//Quantification of IDE localization in human islets

//@author Michael Nelson

//Channels to analyze - in order listed in Brightness and Contrast menu where the first channel is "1"

int FIRST_CHANNEL = 1

int THIRD_CHANNEL = 4

int SECOND_CHANNEL = 2

//Names for export

chn1 = "IDE"

chn2 = "INS"

chn3 = "SST"

//Set background for Manders calculations

ch1Background = 100

ch2Background = 200

ch3Background = 300

//Create objects to be analyzed.

//Requires a classifier/thresholder called INS and SST to have been created for those channels

clearAllObjects()

createAnnotationsFromPixelClassifier("INS", 2.0, 0.0)

createAnnotationsFromPixelClassifier("SST", 2.0, 0.0)

selectAnnotations()

mergeSelectedAnnotations()

makeInverseAnnotation()

def imageData = getCurrentImageData()

def hierarchy = imageData.getHierarchy()

def serverOriginal = imageData.getServer()

String path = serverOriginal.getPath()

double downsample = 1.0

ImageServer<BufferedImage> server = serverOriginal

objects= getAnnotationObjects()

println("Count = "+ objects.size())

//Calculate Pearson and Manders coefficients for channels 1 and 2

objects.each{

//Create bounding box region

roi = it.getROI()

request = RegionRequest.createInstance(path, downsample, roi)

pathImage = IJTools.convertToImagePlus(server, request)

imp = pathImage.getImage()

//Extract the first channel as a list of pixel values

firstChanImage = imp.getProcessor(FIRST_CHANNEL)

firstChanImage = firstChanImage.convertToFloatProcessor() //Needed to handle big numbers

ch1Pixels = firstChanImage.getPixels()

//Create a mask so that only the pixels we want from the bounding box area are used in calculations

bpSLICs = createObjectMask(pathImage, it).getPixels()

//Find number of pixels to be analyzed

size = ch1Pixels.size()

secondChanImage= imp.getProcessor(SECOND_CHANNEL)

secondChanImage=secondChanImage.convertToFloatProcessor()

ch2Pixels = secondChanImage.getPixels()

thirdChanImage= imp.getProcessor(THIRD_CHANNEL)

thirdChanImage= thirdChanImage.convertToFloatProcessor()

ch3Pixels = thirdChanImage.getPixels()

//use mask to extract only the useful pixels into new lists

ch1 = []

ch2 = []

ch3 = []

for (i=0; i<size; i++){

if(bpSLICs[i]){

ch1<<ch1Pixels[i]

ch2<<ch2Pixels[i]

ch3<<ch3Pixels[i]

}

}

size = ch1.size()

if(ch1.size() == 0 || ch2.size() == 0|| ch3.size() == 0){return}

//Calculating the mean for Pearson's

double ch1Mean = ch1.sum()/ch1.size()

double ch2Mean = ch2.sum()/ch2.size()

double ch3Mean = ch3.sum()/ch3.size()

//Calculate the top and bottom parts of the Pearson's coefficient for the "2vs1" and "3vs1" channel comparisons

top2 = []

for (i=0; i<size;i++){top2 << (ch1[i]-ch1Mean)*(ch2[i]-ch2Mean)}

pearsonTop2 = top2.sum()

botCh1 = []

for (i=0; i<size;i++){botCh1<< (ch1[i]-ch1Mean)*(ch1[i]-ch1Mean)}

rootCh1 = Math.sqrt(botCh1.sum())

botCh2 = []

for (i=0; i<size;i++){botCh2 << (ch2[i]-ch2Mean)*(ch2[i]-ch2Mean)}

rootCh2 = Math.sqrt(botCh2.sum())

pearsonBot2 = rootCh2*rootCh1

top3 = []

for (i=0; i<size;i++){top3 << (ch1[i]-ch1Mean)*(ch3[i]-ch3Mean)}

pearsonTop3 = top3.sum()

botCh3 = []

for (i=0; i<size;i++){botCh3 << (ch3[i]-ch3Mean)*(ch3[i]-ch3Mean)}

rootCh3 = Math.sqrt(botCh3.sum())

pearsonBot3 = rootCh3*rootCh1

double pearson2 = pearsonTop2/pearsonBot2

double pearson3 = pearsonTop3/pearsonBot3

String name2 = "Pearson Corr "+":"+chn1+"+"+chn2

String name3 = "Pearson Corr "+":"+chn1+"+"+chn3

it.getMeasurementList().putMeasurement(name2, pearson2)

it.getMeasurementList().putMeasurement(name3, pearson3)

//Start Manders calculations

double m1Top2 = 0

for (i=0; i<size;i++){if (ch2[i] > ch2Background){m1Top2 += Math.max(ch1[i]-ch1Background,0)}}

double m1Bottom2 = 0

for (i=0; i<size;i++){m1Bottom2 += Math.max(ch1[i]-ch1Background,0)}

double m2Top2 = 0

for (i=0; i<size;i++){if (ch1[i] > ch1Background){m2Top2 += Math.max(ch2[i]-ch2Background,0)}}

double m2Bottom2 = 0

for (i=0; i<size;i++){m2Bottom2 += Math.max(ch2[i]-ch2Background,0)}

double m1Top3 = 0

for (i=0; i<size;i++){if (ch3[i] > ch3Background){m1Top3 += Math.max(ch1[i]-ch1Background,0)}}

double m1Bottom3 = 0

for (i=0; i<size;i++){m1Bottom3 += Math.max(ch1[i]-ch1Background,0)}

double m2Top3 = 0

for (i=0; i<size;i++){if (ch1[i] > ch1Background){m2Top3 += Math.max(ch3[i]-ch3Background,0)}}

double m2Bottom3 = 0

for (i=0; i<size;i++){m2Bottom3 += Math.max(ch3[i]-ch3Background,0)}

//Check for divide by zero and add measurements

name = "M1 "+": ratio of "+chn1+" intensity in "+chn2+" areas"

double M1 = m1Top2/m1Bottom2

if (M1.isNaN()){M1 = 0}

it.getMeasurementList().putMeasurement(name, M1)

double M2 = m2Top2/m2Bottom2

if (M2.isNaN()){M2 = 0}

name = "M2 "+": ratio of "+chn2+" intensity in "+chn1+" areas"

it.getMeasurementList().putMeasurement(name, M2)

name = "M1 "+": ratio of "+chn1+" intensity in "+chn3+" areas"

M1 = m1Top3/m1Bottom3

if (M1.isNaN()){M1 = 0}

it.getMeasurementList().putMeasurement(name, M1)

M2 = m2Top3/m2Bottom3

if (M2.isNaN()){M2 = 0}

name = "M2 "+": ratio of "+chn3+" intensity in "+chn1+" areas"

it.getMeasurementList().putMeasurement(name, M2)

}

println("Done!")

//A function that takes the current image and an object, and returns a byteprocessor that is a mask of the current object

def createObjectMask(PathImage pathImage, PathObject object) {

//create a byteprocessor that is the same size as the region we are analyzing

def bp = new ByteProcessor(pathImage.getImage().getWidth(), pathImage.getImage().getHeight())

//create a value to fill into the "good" area

bp.setValue(1.0)

def roi = object.getROI()

roiIJ = IJTools.convertToIJRoi(roi, pathImage)

//fill the ROI with the setValue to create the mask, the other values should be 0

bp.fill(roiIJ)

return bp

}

//Necessary import statements

import qupath.lib.regions.RegionRequest

import ij.process.ByteProcessor;

import ij.process.ImageProcessor;

import java.awt.image.BufferedImage

import qupath.imagej.tools.IJTools

import ij.process.ImageProcessor

import qupath.lib.images.servers.ImageServer

import qupath.lib.objects.PathObject

import qupath.lib.images.PathImage

import qupath.imagej.tools.PathImagePlus
